# Automatic simultaneous determination of copper and lead in biological samples by flow injection/stripping voltammetric analysis

**DOI:** 10.1155/S146392469300015X

**Published:** 1993

**Authors:** Andrés Izquierdo, M. D. Luque de Castro, Miguel Valcárcel

**Affiliations:** Department of Analytical Chemistry Faculty of Sciences University of Córdoba Córdoba E-14004 Spain

## Abstract

An automatic-continuous method for the simultaneous determination of copper and lead based on flow injection analysis (FIA) and stripping voltammetry (SV) is proposed. The method affords the determination of the analytes at the ng/ml level (linear ranges 0.64 to 64.0 ng/ml and 2.1 to 62.2 ng/ml for copper and lead, respectively) with good precision (r.s.d. values smaller than 4%). The selectivity of SV allows the method to be applied to the determination of these analytes in bovine liver fresh samples and certified reference materials from the National Institute for Standards and Technology and the Community Bureau of Reference. The performance of the method was assessed by repeatability and validation statistical studies.


Journal of Automatic Chemistry, Vol. 15, No. 4 (July-August 1993), pp. 121-125

### Automatic simultaneous determination of copper and lead in biological samples by flow injection/stripping voltammetric analysis

**Andrés Izquierdo, M. D. Luque de Castro and Miguel Valcárcel**

*Department of Analytical Chemistry, Faculty of Sciences, University of Córdoba, E-14004 Córdoba, Spain*

*An automatic-continuous method for the simultaneous determination of copper and lead based on flow injection analysis (FIA) and stripping voltammetry (SV) is proposed. The method affords the determination of the analytes at the ng/ml level (linear ranges 0·64 to 64·0 ng/ml and 2·1 to 62·2 ng/ml for copper and lead, respectively) with good precision (r.s.d. values smaller than 4%). The selectivity of SV allows the method to be applied to the determination of these analytes in bovine liver fresh samples and certified reference materials from the National Institute for Standards and Technology and the Community Bureau of Reference. The performance of the method was assessed by repeatability and validation statistical studies.*

### Introduction

Anodic stripping voltammetry (ASV) is a powerful electroanalytical technique for trace metal measurement [1,2]. Various approaches have been proposed for improving its sensitivity: for example by correcting the non-Faradaic charging background current (this involves the application of potential-time wave forms such as differential pulse [3-5], phase selective AC [6], or staircase [7] to replace the conventional linear scan during the stripping step), or by using the most suitable flow-cell (usually thin-layer [4,8] or wall-jet [5,9]).

Continuous stripping methods offer substantial advantages over their batch counterparts [10], in terms of rapidity and sample and reagent consumption. Both flow injection analysis (FIA) [11] and continuous flow methods [12] have been used with automatic stripping voltammetric methods with a clear improvement on both performance and analytical features compared with conventional manual techniques [13]. Few of the reported continuous stripping voltammetric methods used for determination of heavy metals deal with the determination of the analytes in real samples. Of the methods based on SV for copper and lead that have been proposed to date [9,14,15], only one was applied to real samples (atmospheric dust [15]).

The determination of traces and subtraces of metals in biological samples is a growing research area due to the accumulative character of some of them and the restrictive interval and/or ratios of others in living organisms. This paper provides an alternative to the use of atomic spectroscopic methods for heavy metal determinations. The method described is based on a more sensitive, selective and inexpensive technique: ASV. ASV methods for copper and lead were used for the simultaneous determination of both analytes in biological tissues with reference materials and with fresh samples of lyophilized bovine liver.

### Experimental

### *Instruments and apparatus*

Instruments involved included a Metrohm E-505 polarograph connected to a Metrohm 656 wall-jet cell with glassy working electrode, Ag/AgCl (3 M KCl) reference electrode and Au auxiliary electrode, and a Metrohm E-506 stand for degasification were also used. A Gilson Minipuls-3 peristaltic pump, a Rheodyne 5041 injection valve and a Rheodyne 5341 selecting valve were also used to build the FI manifold. A compatible PC with a passive interface, which was built in house, was used for data acquisition and treatment. All potentials were referred to the Ag/AgCl (3 M KCl) electrode. All glass and polyethylene material was kept in 2 M $HNO_3$ for seven days, then rinsed five times with ultrapure water and left in it for one day and finally stored into 0.1 M HCl aqueous solution until use.

### *Reagents*

Aqueous stock solutions of copper and lead of 1 g/l were prepared from nitrates of these cations. Ultrapure water from a Milli-Q plus system (Millipore) was used throughout the experiments. All chemicals used were analytical reagent grade. The carrier and Hg(II) aqueous solutions were degassified by bubbling argon (purity N-50) into the bulk solutions for 10 min. An argon stream was held over the solution surfaces during the experiments in order to prevent any interference from oxygen in the measurement step.

### *Certified reference materials*

Lyophilized bovine liver from the National Institute for Standards and Technology (NIST) (SRM 1577a) and the Community Bureau of Reference (BCR No. 285) and lyophilized pig kidney (BCR No. 186) were used. The instructions for use were followed in each case.

0142-0453/93 $10.00 © 1993 Taylor & Francis Ltd.

*Sample pretreatment*

The wet digestion method proposed by Bond *et al.* [16] was used to dissolve the lyophilized samples and destroy the organic material.

*Manifold and procedure*

Figure 1 shows the flow injection manifold designed to perform the determination, in which in a first step, valve SV selects the Hg(II) stream for conditioning the working electrode surface by deposition of a mercury film. This step applies a potential of −1·0 V to the working electrode for 20 min during which time $1 \times 10^{-4}$ M Hg(II) solution passes through the flow-cell at a flow-rate of 0·2 ml/min. The potential was then switched to 0·0 V and held for 2 min to dissolve any contaminant codeposited with mercury. The system is then ready for preconcentration/determination. For the preconcentration step, SV is switched and the carrier background electrolyte circulates through the system. A deposition potential of −1·0 V is applied to the working electrode; the injection valve is then switched to the inject position thus allowing the injected plug (600 µl) to reach the flow-cell. After passage of the sample plug (3 min of deposition time), the carrier refills the cell and the flow is halted by stopping the peristaltic pump. After 5 s (5 s being the interval necessary to stabilize the solution in the cell) a positive DP scan from −1·0 to 0·0 V (repetition time, 0·4 s; amplitude, 50 mV; scan-rate 40 mV/s) is applied to the working electrode. The oxidation intensity current data are acquired by the computer, which shows the results on the screen after comparing the signals with those provided by the standards used to run the calibration curve of each analyte, which is stored in the management file.

### Results and discussion

The optimization studies included establishing the best working conditions to form the mercury film on the working electrode surface. Optimization of the deposition/stripping step for each analyte was performed separately in order to know the individual behaviour of these, thus facilitating the choice of a common working conditions for the simultaneous determination.

*Conditioning of the working electrode surface*

Aqueous solutions of Hg(II) (from $1 \times 10^{-5}$ to $5 \times 10^{-3}$ M) were continuously circulated through the FI manifold shown in figure 1 and so entered into contact with the glassy carbon working electrode, the features of which depend on the Hg(II) concentration, deposition time and type of waveform (direct current — DC — or differential pulse — DP), and value (from −0·7 to −1·0 V versus the Ag/AgCl reference electrode) of the applied potential. The current intensity generated during each experiment was recorded. A sharp increase of the current intensity was obtained, which reached an almost constant value after 10 min when the surface electrode was covered with a homogeneous film. The electrolysis was continued for 10 min to ensure completion of the process under each working condition. The optimum working conditions to form the mercury film on the electrode surface were:

(1) Concentration of Hg(II): $1 \times 10^{-4}$ M (above this concentration the analytical signal obtained by using the electrode to develop a well-established stripping method kept constant).

(2) Applied potential: −0·9 V (more negative potentials did not improve the analytical signal).

(3) Type of excitation signal: DC (the DP waveform gave place to less reproducible and durable films).

(4) Deposition time: 20 min.

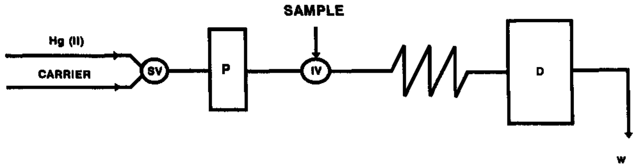

*Figure 1. Flow injection manifold for the simultaneous determination of copper and lead by anodic stripping voltammetry. SV denotes selecting valve; P, peristaltic pump; IV, injection valve; D, detector; and W, waste.*

*Optimization of variables*

The univariate method was used for optimization. From initial values of variables selected according to the literature, the value of one of the variables was changed until its optimal value was found; this was held constant for further experiments. The whole variables were divided into three groups: chemical; FIA; and instrumental variables. The optimal value for each variable is listed in table 1. The optimization of both chemical and FIA variables was very simple because of the absence of previous or parallel chemical reaction and the simplicity of the manifold.

The scarce chemical variables influencing a voltammetric stripping method are the type and concentration of the electrolyte buffer solution acting as carrier in a continuous system, and its pH. The best carrier solution was a 1 M acetic acid/sodium acetate solution of pH 4·5.

Generally, FIA variables have a substantial influence; this dramatically reduced in FI/VS methods as a consequence of the preconcentration step which minimizes the effect of the dispersion along the system. So, variations in the length of the transport tube from the injection system to the flow-cell had a nil effect. The volume of the injected samples is a key variable in stripping methods because the amount of analyte accumulated on the electrode surface is a linear function of the volume of sample which has entered into contact with the working electrode during the preconcentration step. On the other hand the sampling frequency decreases by increasing the volume of injected sample. So a volume of 600 µl was selected as a compromise between sensitivity and sample throughput. The flow-rate strongly affected the sensitivity of the method as high flow-rates afford short electrode/analyte contact time and poor preconcentration factors. An inverse relationship exists between

*Table 1. Optimal values and features of the determination methods for copper and lead.*

| | Variable | Optimal value: Copper | Optimal value: Lead | Common value |
|---|---|---|---|---|
| Chemical | Hg(II) concentration | $1 \times 10^{-4}$ M | | — |
| | Buffer concentration | 1·0 M | | — |
| | $pH_{Buffer}$ | 4·5 | | — |
| Instrumental | Hg plating potential | −1·0 V | | — |
| | Scan rate | 40 mV/s | | — |
| | Pulse amplitude | 50 mV | | — |
| | Deposition potential | −0·9 V | −1·0 V | −1·0 V |
| FIA | Flow-rate | 0·2 mL/min | | — |

| Features of the methods | | | |
|---|---|---|---|
| Parameters | | Copper | Lead |
| Equation | Slope (nA) | $2{\cdot}6461 \times 10^{9} \pm 9{\cdot}2833 \pm 10^{7}$ | $6{\cdot}9258 \times 10^{9} \pm 9{\cdot}4211 \times 10^{7}$ |
| | Intercept (nA) | 10·1754 ± 6·1404 | 65·5640 ± 17·6315 |
| | Regression coefficient | 0·998 | 0·999 |
| Linear concentration range (M) | | $1 \times 10^{-8}$–$1 \times 10^{-7}$ | $1 \times 10^{-8}$–$3 \times 10^{-7}$ |
| RSD (%) | | 3·90 | 1·44 |

flow-rate and peak height, but a decrease in the flow-rate decreased the sampling frequency; so a compromise was also taken in this case by using a flow-rate of 0·2 ml/min.

Prior to optimization of the instrumental variables the differential-pulse waveform was selected for performing the stripping step as the sensitivity achieved by using it is higher than that achievable by the DC scan [4,5]. Other instrumental variables were the rate scan, interval between successive pulses, pulse amplitude and accumulation potential, which were optimized separately for copper and lead. The accumulation potential for each analyte was studied in the range −1·3 to −0·6 V, the best value being −1·2 V. Nevertheless, the use of this deposition potential involved the deposition of traces of zinc contained in the reagents that can be place to the formation of Cu-Zn, $Cu\text{-}Zn_2$ and $Cu\text{-}Zn_3$ intermetallic compounds [17], which are stripped at potentials close to the Cu(II) potential thus increasing the copper signal. So, a deposition potential of −0·9 V was selected to circumvent this shortcoming. Changing of the other instrumental variables had a similar influence on the behaviour of the analytes. Thus, the analytical signal increased with the scan rate, so the faster scan provided by the instrument (40 mV/s) was selected as optimum. The peak height increased for higher pulses, but the reproducibility of the recordings was poorer, so a pulse amplitude of 50 mV was selected as compromise between sensitivity and reproducibility.

### *Features of the methods*

The linear portion of the calibration curve for these analytes was run individually by preparing a series of standards of each. Linear ranges between $1 \times 10^{-8}$–$1 \times 10^{-7}$ M were obtained for both analytes (0·64–64·0 ng/ml and 2·1–62·2 ng/ml for copper and lead, respectively). The repeatability of the methods, established by using 11 different samples in triplicate injection and expressed as percentage of relative standard deviation was 3·9 and 1·4 for copper and lead, respectively. These and other notable and positive figures are listed in table 1.

### *Determination of copper and lead in biological reference materials and samples of bovine liver*

The values of the common variables listed in table 1 were used for the simultaneous determination of the target analytes in reference materials from the NIST and BCR. The method was applied to three different reference material and fresh bovine liver previously lyophilized in the laboratory. Five replicates of each material provided the results listed in tables 2 to 5, which show acceptable

*Table 2. Determination of lead and copper in certified bovine liver (NIST).*

| | 1577a (Bovine liver) | | | |
|---|---|---|---|---|
| | Found value | | Certified value | |
| | μg/g | (%) RSD | μg/g | (%) RSD |
| Pb | 0·148 ± 0·01 | 6·76 | 0·135 ± 0·015 | 11·1 |
| Cu | 177·2 ± 14·7 | 8·28 | 158 ± 7 | 4·4 |

*Table 3. Determination of lead and copper in certified bovine liver (BCR).*

| | No. 185 (Bovine liver) | | | |
|---|---|---|---|---|
| | Found value | | Certified value | |
| | μg/g | (%) RSD | μg/g | (%) RSD |
| Pb | 0·54 ± 0·042 | 7·78 | 0·501 ± 0·027 | 5·39 |
| Cu | 195 ± 5 | 2·56 | 189 ± 4 | 2·12 |

*Table 4. Determination of lead and copper in certified pig kidney (BCR).*

| | No. 186 (Pig kidney) | | | |
|---|---|---|---|---|
| | Found value | | Certified value | |
| | µg/g | (%) RSD | µg/g | (%) RSD |
| Pb | 0·319 ± 0·025 | 7·84 | 0·306 ± 0·011 | 3·59 |
| Cu | 33·6 ± 2 | 5·95 | 31·9 ± 0·49 | 1·53 |

*Table 5. Determination of lead and copper in fresh bovine liver previously lyophilized in the laboratory.*

| | µg/g | (%) RSD |
|---|---|---|
| Pb | 0·146 ± 0·012 | 8·22 |
| Cu | 110 ± 3 | 2·73 |

deviations of the concentrations found as compared with the certified values.

Two statistical studies were carried out to validate the performance of the method. The instructions for use of reference materials from BCR relating to comparison of the results obtained by the proposed method with the values of the certification campaign were used to check the repeatability of the method, which involved the verification that the average of the standard deviations of our results $\left(\frac{s_i}{\sqrt{n}}\right)$ were smaller than the standard deviation(s) of the certified mean. Table 6 lists the results obtained, which are within the limits of a repeatable method, comparable to the analysis performed for certification of these material. To validate the method the BCR recommends assessing whether the mean of the results of the proposed method is within the limits: certified value ±2 s, which corresponds to the interval of the 95% of the population of the laboratory means. Table 7 lists the results obtained by applying the above condition. More than 75% of the results are within this interval.

*Table 6. Repeatability of the proposed method.*

| | | Statistical parameters | | | | | | | |
|---|---|---|---|---|---|---|---|---|---|
| | | $s_i$ | | $\frac{s_i}{\sqrt{n}}$ | | $s$ | | $\frac{s_i}{\sqrt{n}} < s$ | |
| Sample | Reference | Cu | Pb | Cu | Pb | Cu | Pb | Cu | Pb |
| Bovine liver | SRM 1577a | 14·7 | 0·01 | 6·5739 | 0·0045 | 7·0 | 0·015 | Yes | Yes |
| Oyster tissue | SRM 1566a | 6·0 | 0·015 | 2·6832 | 0·0067 | 4·3 | 0·014 | Yes | Yes |
| Skimmed milk powder | BCR No. 150 | 0·3 | 0·07 | 0·1342 | 0·0313 | 0·2 | 0·04 | Yes | Yes |
| Bovine liver | BCR No. 185 | 5·0 | 0·042 | 2·2360 | 0·0188 | 4·0 | 0·027 | Yes | Yes |
| Pig kidney | BCR No. 186 | 2·0 | 0·025 | 0·8944 | 0·0112 | 0·49 | 0·011 | Yes | Yes |

*Table 7. Validation of the proposed method.*

| | | Samples | | | | |
|---|---|---|---|---|---|---|
| Parameter | Analyte | Bovine liver 1577a | Oyster tissue 1566a | Bovine liver No. 185 | Pig kidney No. 186 | Skimmed milk powder No. 150 |
| Certified value | Cu | 158 | 66·3 | 142 | 128 | 2·23 |
| | Pb | 0·135 | 0·37 | 0·501 | 0·306 | 1·0 |
| s | Cu | 7 | 4·3 | 4·0 | 0·49 | 0·18 |
| | Pb | 0·015 | 0·014 | 0·027 | 0·011 | 0·04 |
| Found value | Cu | 177·2 | 70·0 | 195·0 | 33·6 | 2·77 |
| | Pb | 0·148 | 0·38 | 0·540 | 0·316 | 0·984 |
| CV − 2 s | Cu | 144·0 | 57·7 | 181·0 | 30·92 | 1·87 |
| | Pb | 0·105 | 0·342 | 0·477 | 0·284 | 1·92 |
| CV + 2 s | Cu | 172·00 | 74·9 | 197·0 | 32·88 | 2·59 |
| | Pb | 0·165 | 0·398 | 0·555 | 0·328 | 1·08 |
| a < FV< b | Cu | No | Yes | Yes | No | No |
| | Pb | Yes | Yes | Yes | Yes | Yes |

All dates are in µg/g. Corresponding: s, Standard deviation; CV, Certified value; FV, Found value; a, CV − 2 s; b, CV + 2 s; each one.

### Conclusion

The automatic method for the simultaneous determination of copper and lead proposed in this paper allows determination at low levels (well below the limits specified in the international regulations), with simple, inexpensive instrumentation and with results in agreement with the certified values. Better results can be obtained by developing this method in a clean laboratory, which will be justified in terms of costs and time only when special samples are being used.

### Acknowledgement

The authors would like to thank the European Commission for financial support (Contract No. CBM/ST/88-89).

### References

1. Copeland, T. R. and Skogerboe, R. K., *Analytical Chemistry*, **46** (1974), 1257A.
2. Wang, J., *Environmental Science Technology*, **16** (1982), 104A.
3. Copeland, T. R., Christie, J. H., Osteryoung, R. A. and Skogerboe, R. K., *Analytical Chemistry*, **45** (1973), 2171.
4. Wise, J. A., Heineman, W. R. and Kissinger, P. T., *Analytica Chimica Acta*, **172** (1985), 1.
5. Gunasingham, H. and Fleet, B., *Analytical Chemistry*, **55** (1983), 1409.
6. Underkofler, W. L. and Shain, I., *Analytical Chemistry*, **37** (1965), 218.
7. Eisner, U., Turner, J. A. and Osteryoung, R. A., *Analytical Chemistry*, **48** (1976), 1608.
8. Wasberg, M. and Ivaska, A., *Analytica Chimica Acta*, **179** (1986), 433.
9. Tay, E. B., Khoo, S. and Ang, S., *Analyst*, **114** (1989), 1271.
10. Stulik, K., *Analyst*, **114** (1989), 1519.
11. Valcárcel, M. and Luque de Castro, M. D., *Flow Injection Analysis: Principles and Applications* (Ellis Horwood, Chichester, 1987).
12. Goto, M., *Trends in Analytical Chemistry*, **2** (1983), 92.
13. Luque de Castro, M. D. and Izquierdo, A., *Electrolanalysis*, **3** (1991), 457.
14. Wang, J. and Dewald, H. D., *Analytical Chemistry*, **56** (1984), 156.
15. Dabbagh, H. A., Perakis, N., Wolff, C. M. and Schwing, J. P., *Electrochimica Acta*, **29** (1984), 567.
16. Adelojou, S. B. and Bond, A. M., *Analytical Chemistry*, **57** (1985), 1728.
17. Shuman, M. S. and Woodward, G. P. Jr., *Analytical Chemistry*, **48** (1976), 1979.

